# Beyond publishing primary research papers

**DOI:** 10.1128/mbio.02506-24

**Published:** 2024-09-20

**Authors:** Vinayaka R. Prasad

**Affiliations:** 1 Department of Microbiology and Immunology, Albert Einstein College of Medicine, Bronx, New York, USA; Johns Hopkins Bloomberg School of Public Health, Baltimore, Maryland, USA

**Keywords:** minireviews, non-primary research, editorial

## Abstract

Microbiologists, like scientists in any other biomedical field, are too engrossed in writing research papers. Aided by both expanding research programs and shrinking resources, this will continue for the foreseeable time. In this editorial, I discuss a compelling need for all microbiologists to dedicate some time to writing non-research publications such as minireviews, perspectives, commentary, opinion/hypothesis, and other non-research article types. I also list the benefits to the field, of review articles and how they can have the potential to change the field. I have provided a handful of classic examples of reviews that clearly changed the field in a remarkable way as well as a number of reviews that clarified the field and facilitated future research.

## EDITORIAL

Microbiologists, as any biomedical scientists, have a singular focus—making new discoveries that advance the field and disseminating the results via publication of research articles. We occasionally venture beyond this golden path into writing a review, but this tends to be a low-frequency enterprise for most of us, triggered only by a formal invitation, an occasional trainee’s desire to write one, or a colleague’s nudging. However, as I suggest in this editorial, there are additional impactful avenues for publishing beyond writing primary research papers. I discuss how writing review articles can change the field and list other ways in which we could focus our intellectual enterprise to contribute to progress in our respective fields.

### Purpose of writing a review article

As I see it, a primary reason for a scientist to write a review article is to generate a bird’s eye view of the path thus far traveled, conceptual advances made, and the problems solved in a given research area. This exercise, beyond summarizing the work done by a research team and others in the field, also accomplishes other objectives. One, it contrasts the contributions of different groups in the field highlighting commonalities that reaffirm some conclusions while exposing contrasts that require explanations or further work. Two, it helps paint a canvas of the big picture allowing one to identify critical gaps, clarify issues that may be otherwise tangled, and connect ideas from disparate fields. Three, it allows one to lay out a roadmap for future research. Four, perhaps most importantly, it can inspire (or dissuade) other scientists who wish to enter the field and provide a roadmap for them. Reading reviews is also the way I educate myself when I begin working in a new area. Further, reviews can propose new models and discuss new trends and ways of thinking or new hypotheses that revolutionize a field as exemplified by the classic examples mentioned below.

### Classic examples of reviews that changed the field

Below, I offer six examples of reviews that had major impacts on their respective fields. I must hasten to add that even though I have consulted a dozen colleagues for suggestions, ultimately, the select list below is biased and reflects my own interests and knowledge range. Thus, obviously, it forms a very small selection out of a large number of reviews that have changed the field.

#### Mitochondria and chloroplasts as symbionts

A review article in the *Journal of Theoretical Biology* by Lynn Margulis (formerly Lynn Sagan) laid the foundations for testing the idea of mitochondria and chloroplasts as prokaryotic symbionts (1). Sagan not only provided an extensive and in-depth analysis of this idea but was the first to outline a series of testable hypotheses in a well-articulated review. This set the stage for an array of biochemical, morphological, and phylogenetic analyses of eukaryotic nuclei, mitochondria, and chloroplasts that have validated this theory (2).

#### DNA end replication problem

Two review articles, one by Watson (3) and the other by Olovnikov (4), first described the DNA end replication problem. Their astute understanding of the DNA replication mechanism as described in the late 60s led them to predict that the ends of chromosomes are susceptible to terminal erosion, leading to a model that subsequently led to the definition of telomeres and the eventual discovery of telomerase.

#### Pathogen recognition receptors

Another such classic review was one by Janeway outlining the concept of an innate immune system that would recognize any pathogen and foretold the coming discovery of pattern recognition proteins and a whole new class of immunity—the innate immunity (5, 6). Such was the impact of this review that it triggered a race to identify pattern recognition receptors (PRRs) for infecting microbes culminating in the discovery of Toll-like receptors (TLRs) followed by other types of PRRs.

#### Baltimore classification of viruses

In the same breath, we should remember the Baltimore virus classification (7) that was outlined in a review published in *Bacteriological Reviews*. David Baltimore, following his co-discovery of reverse transcriptase and thus updating Francis Crick’s central dogma of molecular biology, outlined six different virus families that could be distinguished from one another on the basis of the different set of steps required for genetic information transfer starting from the virus genome to a +sense RNA (mRNA equivalent) from where the key step of protein synthesis can begin. While being simple, this system played a key role not only in classifying viruses but also in understanding the precise route of genetic information flow. The Baltimore review played a foundational role in virology (8).

#### Human papillomavirus as the cause of cervical cancer

Following his exploration of herpes simplex virus as the potential cause of cervical cancer, it was in a review article that zur Hausen (9) laid out clear arguments for why human papillomaviruses (HPV) may be the underlying cause of cervical cancer. This review appears to have both crystallized his own ideas as well as convinced others in the field that HPV is the likely cause of cervical cancer. This eventually led to the confirmation that HPV causes cervical cancer. Now, HPVs are not only known to cause several cancers but there is also an effective intervention for cervical cancer in the form of HPV vaccination that is saving lives.

#### Quorum sensing

A powerful case for how a really good review can be impactful is found in the history of the discovery of the phenomenon known as quorum sensing in bacteria. This concept first came to light in the mid-1960s from the studies of a Hungarian-born scientist, Alexander Tomasz, on *Streptococcus pneumoniae* (10). Additionally, in 1970, Hastings and Nealson reported that *Vibrio fischeri* could communicate by secreting and sensing a small peptide that served as a signal to switch on bioluminescence (11). Although such primary research reports of this phenomenon as well as a review article (12) were published in the 1960s and 1970s, the review that truly ignited the field was a minireview in the 1990s in the *Journal of Bacteriology* by Fuqua and colleagues where the concept of quorum sensing in bacteria was first defined clearly and a path for future research was laid out (13). I should hasten to add that additional landmark, highly cited reviews (14, 15), as well as foundational studies by Bonnie Bassler and her colleagues, have further buttressed the concept of quorum sensing outlined by the Fuqua et al. review and further accelerated the pace of research on quorum sensing. A PubMed search for the phrase *quorum sensing* yielded over 13,000 hits at the writing of this editorial, of which the Fuqua et al. paper (13) is firmly at the top.

### Reviews that clarified the field

Many microbiologists who are bold thinkers have made a huge contribution to the scientific community by clarifying the field, establishing unifying concepts, and catalyzing the pace of research. I provide three examples that stand out as well as list a number of other such reviews.

#### Mechanism of retroviral recombination

In a memorable review, Coffin (16) offered an incisive analysis of how retroviral DNA synthesis occurs in the infected cells and offered his understanding of how recombination occurs between two copies of viral genomic RNA by a copy choice mechanism at broken ends of retroviral RNA genomes during reverse transcription. Although copy choice recombination was not novel at the time and the retroviral reverse transcription process had been understood in quite some detail by then (17), this review was a product of clarity in thinking and assimilation of several published and unpublished findings on a retroviral replication mechanism at that time. This allowed Coffin to realize that retroviruses overcome single-stranded breaks in their RNA genomes and yet manage to efficiently generate a complete proviral DNA copy via recombination mediated by reverse transcriptase’s ability to switch templates when they encounter a break. Of the multiple competing theories of recombination mechanisms prevalent at that time, it is Coffin’s model that has been experimentally verified (18) to be the one that explains most of the retroviral recombination.

#### Damage–response framework

Casadevall and Pirofski, in a review published 25 years ago, clarified that the concept of pathogenicity and its origins is not merely originating from the direct effects of a pathogen but is also a function of the strength of the immune system’s reaction to the pathogen (19). In this model, an under-reacting or over-reacting immune system is one of the major determinants of the degree of mild or overt pathogenesis. This review also presented six different patterns of damage–response profiles depending on the type and extent of damage and the immune response to it that determined the virulence levels.

#### Phylodynamics of virus transmission

A review article that first introduced the term phylodynamics in the context of RNA virus transmission and evolution landscapes in populations and intra-patient swarms was written by Edward Holmes. This review outlined the different phylogenetic tree patterns that would result when viral infection and spread are influenced by host immunity, availability of susceptible individuals, and availability of vaccines. This review introduced the concept of “phylodynamics” as a model for how immunity, epidemiology, and evolutionary biology shape the relationships of viral sequences in a phylogenetic tree. A number of viruses that cause important human diseases including influenza, HIV-1, HCV, and measles were used as examples. Prior to this report, no one had mentioned the term phylodynamics or outlined how the above factors shape the phylogenetic trees. These concepts were helpful to many virologists and infectious disease biologists who studied the recent SARS-CoV-2 pandemic (20).

### Other reviews worthy of mention

Here, I make a brief mention of a handful of other reviews that have influenced the thinking of the scientific community in impactful ways. This list includes (i) Coffin’s review on HIV-1 viral dynamics, which helped us understand how pre-existing viral variation explains the rapid emergence of drug resistance in HIV-1 (21); (ii) Bieniasz’s review that introduced the concept of intrinsic immunity due to host restriction factors that adds to innate and acquired immunity against HIV-1 (22); (iii) the review by Emerman and Malik pondering how past pathogenic pressures shape the modern repertoire of antiviral responses (23); (iv) the review by Ganser-Pornillos and Pornillos that seamlessly integrated the structural data generated by various groups on TRIM proteins and the structures of HIV-1 capsid/core particles with the biology underlying the primate immunodeficiency virus restriction factor TRIM5a (24); (v) the review by Moriyama et al. on the seasonality of influenza infections; (vi) Schoggins’ review that ponders the function of 10% of human genes that constitute interferon-stimulated genes (25); (vii) García-Sastre’s review that outlines 10 strategies of interferon evasion by viruses (26); (viii) the review by Casanova and Abel on the genetic dissection of immunity to mycobacteria (27); and (ix) the review by Raposo and Stoorvogel, which introduced the conceptual framework around and a means of experimentally characterizing extracellular vesicles (28).

### Other benefits

Preparing and publishing a review article that presents the work done by one’s lab over a few years can benefit their current and future trainees. For a starting graduate student or a post-doctoral fellow in a laboratory, even being a co-author on an article that reviews the work done so far can be an amazing groundwork ahead of starting one’s own research in that project. For finishing graduate students, it could be a way to publish a more refined and advanced version of their introductory chapter in their doctoral thesis (which is already a publication) to become a published work in addition to being a morale booster adding yet another paper to their personal bibliography.

### Can anyone write an *mBio* minireview article?

Yes, in theory. In practice, however, the editors and readers would like to know the perspectives of someone who is a practitioner of the science. Graduate students or post-doctoral fellows working in a field can absolutely write a minireview article. But they should do so in close collaboration with their faculty advisor (or other experts in the field) who has a longer history in following the progress in a field. Indeed, senior investigators bring perspective, experience, and a seasoned approach and will be able to guide a less experienced graduate student to navigate the process of writing the review article.

### Ways to publish in *mBio*

The minireviews in *mBio* are just one major type of non-research articles that can make an impact on the research in a given field. *mBio* also offers other means of shaking the tree to let nuggets fall to the ground. *mBio* offers Perspectives, which are brief reviews that offer a succinct overview of a specific topic with an emphasis on opinion and synthesis. mGems, a forthcoming new type of Perspective article, will be an ongoing collection of brief and succinct reviews on topics of current interest within the field of microbiology. If you have a novel or unique opinion about a specific problem, a new direction that the field could take, or a new approach, you could write a short Opinion/Hypothesis article. Opinions/Hypotheses are short articles with original and well-developed insights without complete supporting data. This type of article allows you to publicize a new thought that is formulated in a manner that summarizes a problem, provides a new synthesis, and/or is suitable for further experimental testing. Perhaps you wish to write an Observation—Observations are short descriptions of research results of exceptional importance and unusual interest to the broad microbiology community. Commentaries are short articles that discuss issues of particular significance to the field of microbial sciences. Commentaries may discuss papers of special interest published in *mBio* or other timely information or comment. There are other article formats such as Matters *Arising* and Letters to the Editor as well. See here for more information on formatting your manuscripts, word counts, and other essential guidance: mBio Formatting Information. See Table 1 for a list of various article types published by *mBio* and the type of content we wish to see in each of them. I urge you to use these various article types to voice your thoughts among the community of your peers to tap into the aforementioned benefits.

**TABLE 1:** Types of articles published by *mBio*

Article type	Main feature	Needs data included
Research Article	A research report	Yes
Minireview	Reviews a field	No
Opinion/Hypothesis	Original insights without complete supporting data	No
Observation	Short description of research results of exceptional importance	Yes
Commentary	About an *mBio* paper or something important to field	No
Perspective	Brief review that offers a succinct overview of a specific topic with an emphasis on opinion and synthesis	No
mGem	A type of Perspective that focuses on a current topic and is written for a broad audience. mGems appear on a featured collection page on the *mBio* website	No
Editorial	Communicated by members of the *mBio* Board of Editors	No
Letter to the Editor	Comments on articles published in *mBio*	Yes/no
Matters Arising	A means to alert the readership that the conclusions published in an ASM journal require re-examination	Yes/no
